# Electrochemical Impedance Spectroscopy Analysis of Hole Transporting Material Free Mesoporous and Planar Perovskite Solar Cells

**DOI:** 10.3390/nano10091635

**Published:** 2020-08-20

**Authors:** Sumayya M. Abdulrahim, Zubair Ahmad, Jolly Bahadra, Noora J. Al-Thani

**Affiliations:** 1 Center for Advanced Materials (CAM), Qatar University, Doha 2713, Qatar; sumayya@qu.edu.qa; 2 Qatar University-Young Scientists Center (QU-YSC), Qatar University, Doha 2713, Qatar; jollybhadra@qu.edu.qa (J.B.); n.al-thani@qu.edu.qa (N.J.A.-T.)

**Keywords:** HTM-free PSCs, nanostructures, mesoscopic, impedance spectroscopy, electrical equivalent circuit, power conversion efficiency

## Abstract

The future photovoltaic technologies based on perovskite materials are aimed to build low tech, truly economical, easily fabricated, broadly deployable, and trustworthy solar cells. Hole transport material (HTM) free perovskite solar cells (PSCs) are among the most likely architectures which hold a distinctive design and provide a simple way to produce large-area and cost-effective manufacture of PSCs. Notably, in the monolithic scheme of the HTM-free PSCs, all layers can be printed using highly reproducible and morphology-controlled methods, and this design has successfully been demonstrated for industrial-scale fabrication. In this review article, we comprehensively describe the recent advancements in the different types of mesoporous (nanostructured) and planar HTM-free PSCs. In addition, the effect of various nanostructures and mesoporous layers on their performance is discussed using the electrochemical impedance spectroscopy (EIS) technique. We bring together the different perspectives that researchers have developed to interpret and analyze the EIS data of the HTM-free PSCs. Their analysis using the EIS tool, the limitations of these studies, and the future work directions to overcome these limitations to enhance the performance of HTM-free PSCs are comprehensively considered.

## 1. Introduction

A complete understanding of PSCs has turned out to be a challenge due to their dynamic behavior and complex multidimensional nanostructure. Recently, electrochemical impedance spectroscopy (EIS) has emerged as a useful tool in breaking down the complex dynamic processes occurring within the different layers of solid-state PSCs, which makes it easier to analyze the interfaces and charge dynamics of this type of solar cells. EIS technique has proven to be an effective tool in analyzing the processes occurring at the interface of different layers of the PSC [1,2]. For instance, Bernal et al. [3] qualitatively and quantitatively assessed the different dynamics processes in the PSC by EIS, while Klotz et al. [4] also used EIS results to assess charge separation and recombination processes and related them with a temporary loss in an active area of the device due to single grains altering the perovskite layer. Similarly, the EIS tool has been used consistently in previous works for several other kinds of analyses such as comparing 2D/3D PSCs with 3D PSCs [5,6,7], stability analysis [8,9], studying charge dynamics [4,10], studying the effect of different conditions and architecture on the performance of PSCs [11,12], etc. The frequency-dependent spectra allow to breakdown the different dynamic processes occurring at each interface or bulk with distinguished time constants. Hence, with the help of this tool, the layers responsible for the degradation in the PSCs can be identified by assessing the behavior of each layer over time.

Different architectures of PSCs have been studied and analyzed by the EIS technique in previous works. This includes the conventional n-i-p structure [11] and the inverted p-i-n structure [13]. In addition, researchers have also explored ETL-free [14,15,16] and HTM-free PSCs [17,18,19,20]. This review is focused on the HTM-free PSCs since they have gained increased attention in the solar energy field due to their cost-effective and simple fabrication procedure. A wide variety of perspectives has been adopted by researchers to enhance the performance of HTM-free PSCs such as exploring different counter electrodes [17,18,20,21,22,23,24,25,26,27,28,29,30,31,32,33,34,35,36,37,38,39,40,41,42,43,44] and manipulating the composition of the counter electrodes itself [17,20,24,25]. Besides, the inverted HTM-free PSCs have also been explored. This review summarizes a wide range of these approaches and their progress.

This review provides an insight into the EIS technique and how it is essential to understand the complex behavior of HTM-free PSCs better. EIS analysis has been discussed based on Nyquist and Bode plots, which are the most common methods of analyzing EIS data. The relevant physical parameters that can be extracted from the EIS data by equivalent circuit modeling are also discussed. Furthermore, a comprehensive literature review on EIS analysis employed in previous works was also performed to analyze the trend of utilizing this tool in the field of HTM-free PSC research over the past years.

## 2. Progressions in HTM-Free PSCs

**Figure 1:**
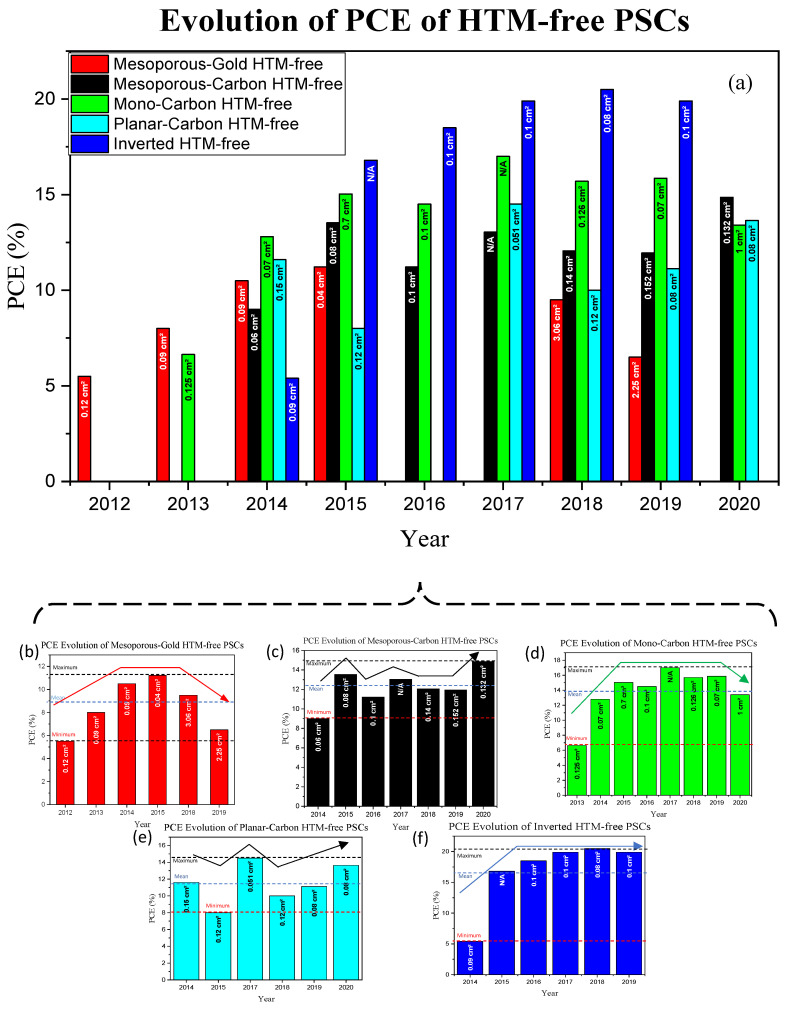
(**a**) Evolution of PCE (power conversion efficiency) of different types of HTM-free PSCs; Gold (Au) counter electrode (CE)-based, mesoporous carbon-based (meso-carbon HTM-free), monolithic carbon-based (mono-carbon HTM-free), planar carbon CE-based (planar-carbon HTM-free) and the inverted HTM-free PSC. The active area of the fabricated devices is mentioned in the bar-charts (N/A is denoted for area values that are not mentioned in the published file). The data are obtained starting from 2012 (when HTM-free PSC was first introduced, with a gold counter electrode). The individual progress of the different architectures can also be observed in (**b**–**f**).

Figure 1 represents the power conversion efficiency (PCE) of the different types of HTM-free PSCs (since the year of the first report) and their active areas considered during the efficiency measurements. The PCE values of each year were plotted based on the highest efficiencies achieved in the respective year. Au counter electrode (CE) was first introduced in HTM-free PSCs; however, not much research has been done with Au CE since it increases the production cost, and its deposition process is complex as well. Subsequently, the research on carbon CE in HTM-free PSCs also started as a low-cost alternative. Specifically, considerable attention has been paid to the monolithic design of the carbon-based mesoscopic PSCs due to its high potential for large-size and commercial-scale production. Figure 1d shows that the PCE of this architecture increased tremendously within the past few years, and then saturated to approximately 15 ± 2%; however, a minor decrease in the PCE value is observed for 2020, which is due to the larger active area of the fabricated device. Limited research has also been performed on the carbon-based planar HTM-free PSC architecture, and it has shown great potential with its commonly employed low temperature processed compact layer, SnO_2_, beneficial for commercialization, and impressive PCEs. The overall PCE of carbon-based planar HTM-free PSCs is increasing, considering the active area (Figure 1e). The PCE of the inverted architecture was initially lower (in 2014) as compared to the other designs. However, its performance soon surpassed the other architectures and saturated to approximately 19 ± 1%. However, despite the higher PCEs achieved with the inverted structure, its high efficiency is reported over a very active but small area. The detailed evolution of the different architectures of HTM-free PSCs is described in the next section.

## 3. Advancements in Different Architectures of HTM-Free PSC

**Figure 2:**
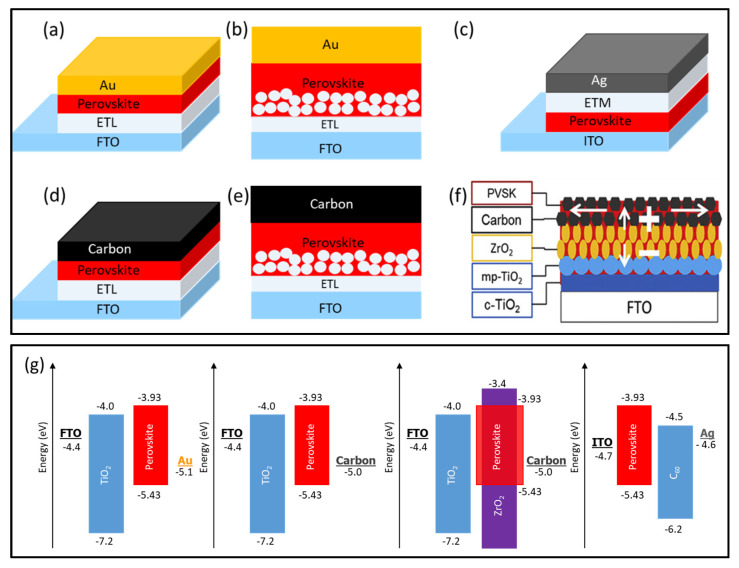
Schematic illustration of commonly employed HTM-free perovskite solar cell structures: (**a**) Planar architecture with Au as the back electrode; (**b**) mesoporous architecture with Au as back contact; (**c**) planar configuration with Ag as the rear electrode; (**d**) planar architecture with carbon as back contact; (**e**) mesoporous structure with carbon as back contact; and (**f**) monolithic device with carbon as the counter electrode. Reproduced with permission from [45]. Elsevier, 2018. (**g**) Schematic illustration of energy band diagrams of p-i-n and n-i-p HTM-free PSC structures with different counter electrodes. The key difference between mesoporous and monolithic carbon-based architectures is the perovskite deposition technique. In monolithic structure, the perovskite is infiltrated into the device after the deposition of all layers (see Figure 3c), whereas the perovskite layer is deposited over the mesoporous TiO_2_ layer before the carbon layer in the mesoporous architecture (there is no spacer layer in this design).

Some commonly employed HTM-free PSC structures in previous works are shown in Figure 2a–f. We categorize these configurations into two different types: (i) with metal top electrodes; and (ii) carbon-based HTM-free configuration. The corresponding energy band diagrams are given in Figure 2g. It can be observed that there is a very small difference in the work function of carbon (−5.0 eV) and Au (−5.1 eV), making carbon an ideal choice to replace Au as the counter electrode and a fully printable HTM-free PSC structure (monolithic PSC) can be fabricated [37]. In the next section, we describe the developments related to each architecture one by one.

### 3.1. HTM-Free PSCs Based on Au Counter Electrode

A very compatible work function of Au with perovskite makes it an excellent choice as the back contact for HTM-free PSCs (see Figure 1g). There are two main designs (mesoporous and planar) that have been reported for the Au counter electrode-based HTM-free devices, which are described below.

#### 3.1.1. Mesoporous Architecture

**Figure 3:**
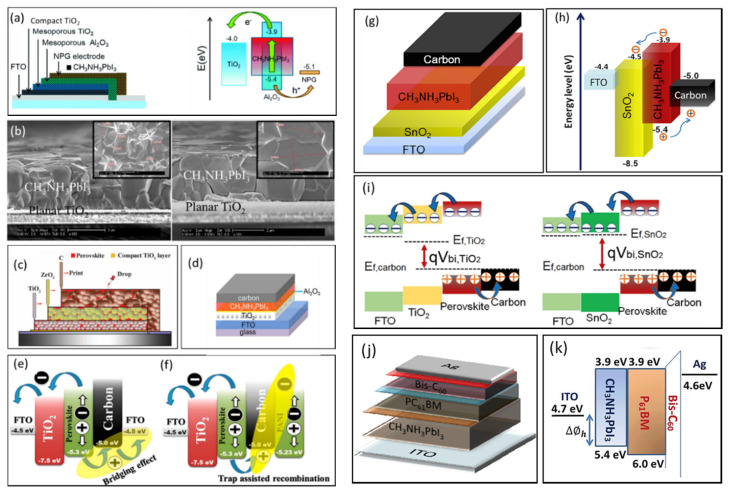
Schematic illustration of the different approaches developed by researchers for HTM-free perovskite solar cell architectures (**a**) with the nonporous gold counter electrode and its corresponding energy bandgap diagram. Reproduced with permission from [26]. Royal Society of Chemistry, 2015. (**b**) Cross-section scanning electron microscopy (SEM) for 8 (left) and 10 (right) perovskite spray passes. Reproduced with permission from [24]. American Chemical Society, 2015. (**c**) Monolithic PSC with perovskite infiltrated in the stacked structure. Reproduced with permission from [38]. American Association for the Advancement of Science, 2014. (**d**) Mesoporous PSC with carbon layer on top of screen printed perovskite layer, and Al2O3 spacer layer. Reproduced with permission from [36]. Wiley-VCH Verlag GmbH & Co. KGaA Weinheim, 2019. (**e**) Schematic of energy band diagrams of FTO-based PSC and (**f**) polyaniline-based PSC, showing the bridging and trapping sites. Reproduced with permission from [46]. Elsevier, 2015. (**g**) Schematic illustration of carbon-based planar PSC with SnO_2_ as the ETL and (**h**) the corresponding energy band diagram. Reproduced with permission from [39]. Elsevier, 2019. (**i**) Schematic illustration of the energy band diagram of planar HTM-free PSCs using TiO_2_ and SnO_2_ as the ETLs, showing their fermi levels. Reproduced with permission from [47]. Elsevier, 2018. (**j**) Schematic illustration of inverted HTM-free structure with PCB61M as the ETL and (**k**) its corresponding energy band diagram. Reproduced with permission from [48]. Royal Society of Chemistry, 2015.

Etgar et al. [20] introduced mesoscopic perovskite/TiO_2_ heterojunction HTM-free PSC for the first time (in 2015) utilizing gold as the back contact and achieved a PCE of 5.5%. The Au layer was thermally evaporated onto the cell. Since then, various modifications have been performed by researchers to optimize it further. Laban et al. [17] further optimized HTM-free PSC by depositing a thick perovskite film by spin coating, in a two-step process and used the thermally evaporated Au the counter electrode. The J–V measurements showed a depletion region at the perovskite/TiO_2_ junction, which aided in charge separation and prevented the recombination of electrons and holes and hence provided a PCE of up to 8%. Later on, to avoid the complex vacuum and energy-intensive deposition process of Au, Zhou et al. [26] fabricated a directly transferrable nanoporous structured gold electrode with a de-alloying method which makes the fabrication a little simpler. The schematic of the employed HTM-free structure is illustrated in Figure 3a. The nanoporous gold structure proved to be an excellent choice as the counter electrode in HTM-free PSCs due to its high conductivity combined with high surface area and stability. Moreover, they also analyzed three different deposition techniques: one-step spin coating, sequential deposition, and two-step spin coating for the fabrication of perovskite on the device performance. The different techniques were analyzed by XRD patterns, and the results show that the two-step spin coating method produced weaker intensity of the characteristic peaks, and hence it was concluded that the one-step spin coating and sequential deposition technique produced better crystallinity of the perovskite. However, the SEM images of the two-step spin-coating method showed better infiltration into the porous structure and higher contact area between the nanoporous Au and perovskite. Moreover, the PCE obtained using this technique was 7.99%, which was the highest among the methods used. In addition, the role of Al_2_O_3_ in HTM-free PSC was also studied, and it was reported that the presence of Al_2_O_3_ increases the PCE because it prevents the recombination of electrons and holes at the TiO_2_ and nanoporous Au interface. Shi et al. [18] also described an HTM-free PSC with Au as the counter electrode and achieved a PCE of 10.49% and further clarified the working mechanism of HTM-free PSCs. I–V characterization model was used to analyze the ideality factor, and series resistance of HTM-free PSC and the values were compared with a typical heterojunction solar cell whose ideality factor is usually in the range of 1.3–2. Their work termed that HTM-free PSC is indeed a heterojunction solar cell and not a sensitized cell. Later on, the PCE of HTM-free PSCs was further enhanced to 10.85% by Aharon at al. [21], who revealed that the performance of HTM-free PSC strongly depended on the width of the depletion layer at the perovskite/TiO_2_ junction which can be estimated by Mott Schottky analysis. Furthermore, it was also analyzed that the width of the depletion layer could be controlled by the thickness of the mesoporous TiO_2_ film. It was observed that at 620 nm± 25 nm thickness, more than half of the TiO_2_ film was depleted, and hence the PCE obtained was the highest among the samples.

#### 3.1.2. Planar Architecture

Initial work on planar HTM-free PSCs with Au back contact was performed by Gamliel et al. [24], who deposited perovskite with the spray coating technique to produce micrometer perovskite crystals. The perovskite precursor was sprayed on hot substrates where the DMF evaporated immediately, creating perovskite crystals. The perovskite film thickness was controlled by the number of spray passes. It was concluded that ten passes created a film thickness of 3.4 μm, which gave the highest PCE of 6.9% (Figure 3b). This spray coating technique of perovskite is not suitable for device architectures containing mesoporous metal oxides due to the percolation of perovskite grains into the mesoporous layers. Therefore, the probability of electron–hole recombination increases, and hence the device performance is affected. Utilizing Au back contact in planar HTM-free architectures has not gained much attention and thus needs further research to explore the potential of using the gold counter electrode in this type of PSC configuration.

### 3.2. HTM-Free PSCs Based on Carbon Counter Electrode

Besides an expensive energy-intensive vacuum deposition process of Au, it has also been reported that the Au could be a potential reason behind the degradation of the PSC devices by diffusing into the perovskite layer [49]. Subsequently, carbon is a much cheaper and more stable material to utilize as a replacement for the Au counter electrode. It also avoids the highly energy consumptive process of vacuum deposition of Au. Moreover, it is also hydrophobic, inert to ionic migration [50], and it is available in abundance allowing for large-scale and economical production.

#### 3.2.1. Monolithic Architecture

The research on carbon-based HTM-free PCS is mostly concentrated on monolithic architectures. Han’s group provided a significant contribution to carbon-based HTM-free PSCs research, and they introduced a unique type of mesoporous HTM-free PSC; monolithic PSC, where all the layers are screen printed and stacked, as illustrated in Figure 3c. The perovskite is then infiltrated into the device in the end, by drop-casting technique. It is a cost-effective, fully printable PSC allowing for large-scale production [37]. They employed a carbon/graphite electrode in HTM-free PSCs for the first time and initially achieved a PCE of 6.7%. Flaky graphite and spheroidal graphite were compared with the I-V characterization technique, incident photon to current conversion efficiency (IPCE) spectrum, and scanning electron microscopy (SEM). It was reported that the spheroidal graphite produced a better performance with a higher PCE. Later on, they improved the PCE of HTM-free PSC to 10.64% by utilizing TiO_2_ nanosheets [40]. The improved PCE was attributed to the high reactivity of exposed facets in TiO_2_ nanosheets, which enhanced the interfacial properties between the compact TiO_2_ and perovskite. Afterward, over 11% of PCE was achieved by optimizing the size of the graphite/carbon counter electrode [43]. The influence of different sized counter electrodes was analyzed by impedance spectroscopy, and it was determined that a thickness of 9 μm provided the best performance. The PCE was further enhanced later on, to 12.8% for a fully printable mesoscopic HTM-free PSC, which was stable for more than 1000 h under illumination conditions [38]. A double mesoporous layer of TiO_2_ and ZrO_2_ was employed and covered by a porous carbon layer. 5-AVA additives were also incorporated to improve the PCE. In later research, ammonium chloride was incorporated into the perovskite precursor, which increased the PCE to 15.6% [51].

#### 3.2.2. Mesoporous Architecture

Significant research on carbon-based HTM-free PSCs has been dedicated to mesoporous configurations. The multiple mesoporous layers in this type of architecture prevent the recombination of electrons and holes and also avoids pure ohmic shunts [52]. Bhatt et al. [46] fabricated an HTM-free mesoscopic carbon-based PSC in ambient air, where polyaniline on FTO was used as a current collector electrode. It was revealed that the PCE increased by 21% with a polyaniline electrode relative to the FTO electrode. However, the V_oc_ was observed to be decreasing. This phenomenon was explained by a new approach, known as bridging and trapping effects. By analyzing the energy band diagrams illustrated in Figure 3e–f, it was revealed that the carbon layer in FTO-based PSC enhances charge transfer at perovskite/TiO_2_ interface due to bridging effect. Consequently, in polyaniline-based PSC, the carbon layer causes charge trapping due to mismatching energy levels of perovskite and polyaniline electrode resulting in trapping effect, and hence the V_oc_ decreases. Ke et al. [36] also reported a carbon-based mesoporous HTM-free PSC fabricated in ambient air with a PCE of 10.7%. The performance was enhanced by incorporating tetrahydrofuran (THF) in the perovskite precursor to produce a uniform film. Al_2_O_3_ mesoporous layer was also incorporated between perovskite and TiO_2_ layers to avoid direct contact, and hence electron–hole recombination was suppressed. The fabricated device is illustrated in Figure 3d.

#### 3.2.3. Planar Architecture

This type of architecture does not include mesoporous scaffold and insulating layers, as shown in Figure 2d and has carbon as the back counter electrode, which makes it a simpler and low-cost architecture. Lv et al. [50] fabricated a fully air-processed planar HTM-free PSC with a PCE of 11.12% where air-stable perovskite; CsPbBr_3_ was used, and carbon and TiO_2_ were used as the back counter electrode and ETL, respectively. Most of the research on this type of architecture is concentrated on low temperature processed SnO_2_, such as the ETL illustrated in Figure 3g,h, instead of the most commonly employed compact and mesoporous layers of TiO_2_, which require high temperature for processing and sintering. SnO_2_ has been consistently used in previous works as the ETL in HTM-based PSCs and has shown impressive electrical and chemical properties with a wide bandgap and high charge mobility [53] and has achieved PCEs up to 20%. Therefore, researchers have attempted to utilize this ETL in HTM-free architectures as well, to obtain a lower fabrication cost-based solar cell beneficial for commercialization. Lin et al. [47] were the first ones to report a low-temperature processed ETL, SnO_2_ in a planar HTM-free PSC architecture, and achieved a PCE of 14.5%. This device was compared with the conventional high temperature processed TiO_2_-based HTM-free PSC device, and it was observed that the V_oc_ of SnO_2_ (1.07 V)-based device was 100 mV higher than that of TiO_2_-based PSC. This is due to SnO_2_’s impressive hole blocking and electron transporting ability which is attributed to its enhanced fermi level owing to its wider bandgap, as illustrated in Figure 3i. This results in a higher Vbi (built in potential), according to Mott–Schottky study. Therefore, the V_oc_ increases due to inhibition of charge recombination. Recently, Vijayaraghavan et al. [53] fabricated a low temperature processed fully printable HTM-free PSC using SnO_2_ quantum dot as the ETL and achieved a PCE of 13.6%. SnO_2_ quantum dots were reported as superior to other solution-processed SnO_2_ and colloidal nanoparticles-based SnO_2_ due to their long-term stability, higher molar extinction coefficient, fast electron extraction, and hole blocking property, which results in an enhanced PCE.

Planar carbon-based HTM-free PSCs are not very popular because the mesoporous scaffold and spacer layers, like in mesoporous and monolithic architectures (Figure 2e,f) are normally required between the front and back contact of the HTM-free PSCs to keep them separated and prevent ohmic shunt. Otherwise, the probability of electrons holes recombination becomes high; however, researchers have overcome this issue by exploring excellent ETL candidates with efficient hole extraction and electron transport abilities and have achieved moderate PCEs with these types of PSCs.

### 3.3. Inverted HTM-Free PSCs (with Ag Counter Electrode)

Most of the work on the inverted structure utilizes silver (Ag) as the back metal electrode instead of Au. Ag usually is not used in the standard architecture since the iodine in the perovskite can react with Ag. However, the inverted structure makes it possible to utilize Ag as the metal electrode. This architecture also avoids the need for mesoporous metal oxide layers such as TiO_2_ and Al_2_O_3_, which require high temperature (400–500 °C) for deposition. Hu et al. [54] were the first to propose inverted HTM-free PSC with the configuration of ITO/CH_3_NH_3_PbI_3_/C_60_/Ag, and a PCE of 5.4% was achieved with a sequential vapor deposition technique for the growth of highly uniform perovskite film. The perovskite was deposited directly on the ITO substrate. Since then, impressive progress has been made on inverted HTM-free PSC research. Tsai et al. further enhanced the PCE to above 11% with an impressive open circuit voltage of 1.1 V by fabricating an HTM-free perovskite/fullerene heterojunction PSC with the configuration of ITO/MAPbI_3_/PC_61_BM/bis-C_60_/Ag, as illustrated in Figure 3j,k. They discovered that perovskite is responsible for altering the work function of ITO, resulting in an enhanced charge extraction efficiency at perovskite/ITO interface. Later on, the PCE was further increased to 16% by Li et al. [27], who fabricated HTM-free PSC by the solution process. The PCE was also improved to 18.1% with almost no I-V hysteresis, by Ye et al. [55] with the configuration of ITO/MAPbI_3−x_Cl_x_(CuSCN)/C_60_/BCP/Ag. CuSCN was incorporated in the perovskite to form a bulk heterojunction, where CuSCN played the role of hole conductor. Later on, there was a major breakthrough by Wu et al. [56], who managed to achieve a PCE surpassing 20% for HTM-free PSCs with a configuration of ITO/MAPbI_3_:F4TCNQ/C_60_/BCP/Cu, by molecular doping of the perovskite film which improved its conductivity and its contact with the substrate. This resulted in a reduced series resistance and an enhanced PCE. Moreover, the doctor-blading deposition technique was acquired, enabling a scalable production.

## 4. Perovskite Solar Cells and Impedance Spectroscopy

### 4.1. The Fundamental Concept of IS and PSCs

**Figure 4:**
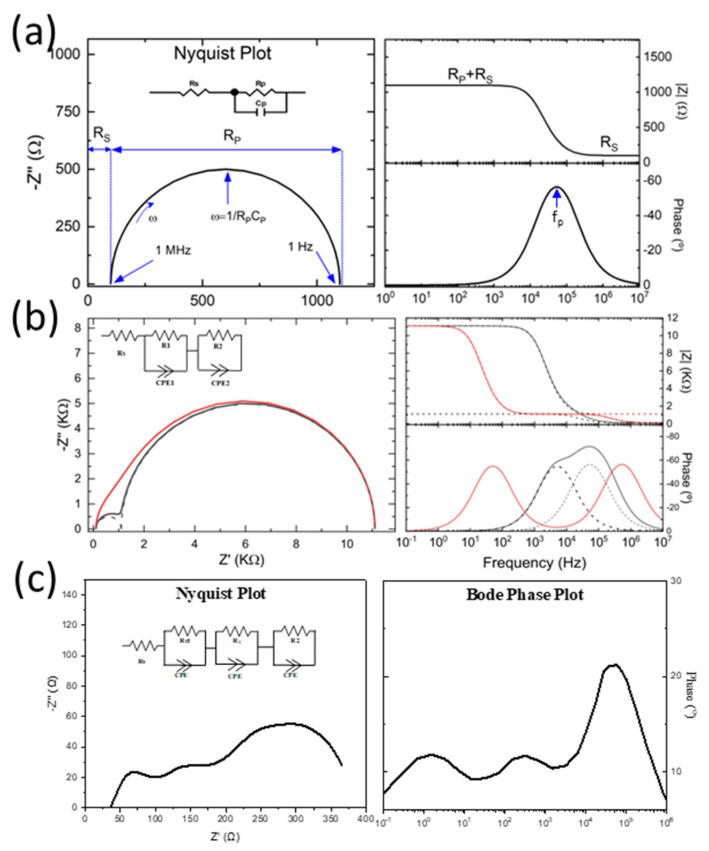
Representation of Impedance spectra using Nyquist plots (left) and Bode plots (right): (**a**) 1-RC circuit; (**b**) 2-RC circuit, black lines represent the Nyquist and Bode plots with well-separated arcs while the red lines symbolize the merged arcs (Reproduced with permission from [59]. Society of Photo-Optical Instrumentation Engineers, 2019.); and (**c**) 3-RC circuit. The corresponding RC circuit diagrams can be found in the inset of Nyquist plots.

Electrochemical impedance spectroscopy (EIS) refers to a technique that is utilized to gain insight into the bulk and interfacial properties of multijunction devices, and it can be used to study the devices under different in-situ conditions such as a function of different variables such as dc voltage, illumination intensity and temperature [57]. It has been progressively applied to perovskite solar cells (PSCs) by researchers over recent decades to study the behavior of PSCs as a function of multiple dynamic processes under different operating conditions. EIS provides the impedance response of the sample against a wide frequency range, usually 0.1–1 MHz against an AC applied potential. The EIS data are commonly interpreted by a Nyquist plot with Z imaginary and Z real along the *y*-axis and *x*-axis, respectively. The resultant spectra can exhibit one, two, three, or more semicircles (depends on the time constants within the applied frequency range). Each arch can be represented by an RC element in an electrical equivalent circuit (EEC) model. Figure 4 illustrates some commonly obtained Nyquist plots with their corresponding Bode plots, and the RC circuit diagrams can be observed in the inset of Nyquist plots. Figure 4a is the impedance spectrum of one RC element. The corresponding frequency data can be extracted from the Bode plot. The maximum of the semicircle; polarization resistance (Rp) in the Nyquist plot represents the peak frequency (fp) in the Bode plot. The corresponding highest and lowest frequency values are marked in the Nyquist plot of Figure 4a. Figure 4b illustrates the impedance spectra of two RC elements. When more than one RC feature is present, the semicircles obtained can be well separated or overlapped. The separated and overlapped semicircles can be defined by τ, which is a time constant representing relaxation time scales. If the time scales of the different dynamic processes are discrete, then separated semicircles are observed. Consequently, when the relaxation time scales are indistinct, the semicircles are overlapped or merged, and it becomes complex to analyze the data. Figure 4c illustrates the impedance spectra of three RC elements, where three clear semicircles at high frequency (HF) (>10^4^), intermediate frequency (IF) (10–10^4^ Hz) and low frequency (LF) (<10 Hz) regions are evident, and these arcs are formed by a parallel combination of resistance and interfacial capacitance [58].

The HF region represents geometric capacitance, which is commonly provided by the carbon or gold electrode in HTM-free PSCs. The IF arc represents the recombination process in the mesoporous TiO_2_/perovskite interface or in the bulk of these layers. The LF region is related to ionic motion as well as recombination processes, and it is very complex to analyze due to hysteresis related to ionic motion [60]. Sometimes, negative loops also appear, which has stirred a high debate on the interpretation of its origin. It has been argued that they are due to the charge accumulation at the interfaces or bulk of semiconducting layers [61,62,63]. Ebadi et al. [64] explained negative capacitance to be just a result of hysteresis. Electrical equivalent circuit (EEC) modeling is the most common tool used to interpret the EIS data physically.

### 4.2. Electrical Equivalent Circuit Modeling

**Figure 5:**
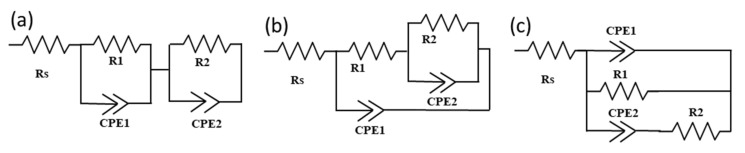
Electrical equivalent circuits used for impedance spectra modeling representing two-time constants in the Nyquist plot: (**a**) Voight circuit; (**b**) ladder-type circuit (Matryoshka); and (**c**) Maxwell circuit.

There are different configurations of electrical circuits that are employed for the fitting of the EIS data (as mentioned in Figure 5), i.e., series type circuit also known as Voight circuit, ladder (or Matryoshka) type circuit, and Maxwell (dielectric) circuit. The ladder-type circuit represents multiple processes occurring simultaneously with different characteristic times [59]. Most commonly, a combination of ladder-type and Voight type configuration is employed for the PSCs. The Maxwell equivalent circuit provides a more complex relationship with the EIS data. Todinova et al. [60] reported that the Matryoshka, Voight, and the mixed Matryoshka–Voight circuit produces equally good fitting of the EIS data. Therefore, one can equally choose between these models. In contrast, the Maxwell circuit may provide values that differ from the empirical EIS data. It should be noted that impedance spectra do not generally exhibit a perfect semicircle, and they are usually flattened due to non-ideal capacitances. Therefore, the EIS data are modeled using a constant phase element (CPE) instead of an ideal capacitor. CPE = −1 represents inductance, CPE = 0 represents resistance, and CPE = 1 represents pure capacitance. The R_ct_ that appears in the high-frequency region is typically attributed to the counter electrode, carbon or gold electrode, and their interface with the perovskite layer (in the case of PSCs). Another resistance element is required to represent the charge transfer at the electron transport layer (ETL) and perovskite interface in the lower frequency region. This is commonly the case when only two arcs appear in the Nyquist plot, [28,65,66]. In this case, 2-RC element equivalent circuit models are required, as illustrated in Figure 5. This is usually the case at low potentials were the processes at the mesoporous layers are not evident [28,67]. At low potentials, the mesoporous layers in the PSCs act as insulators, and hence the recombination resistance is very high and merges into the curve. However, at higher potentials, the mesoporous layers start behaving as conductors or semiconductors (depending on applied potential value) and hence their time constant is evident in the Nyquist plot, sometimes as a small arc in the low-frequency region or as a negative capacitance or as an appearance of an origin of negative capacitance [67]. Hence, the interpretation of the EIS data using the EEC analysis becomes very complicated due to multi-interfacial and ion diffusion processes. Moreover, different circuit configurations are required when analyzing different potentials, since the behavior of PSC depends strongly on the applied potential.

### 4.3. Proposed EEC Models for HTM-Free PSCs

**Figure 6:**
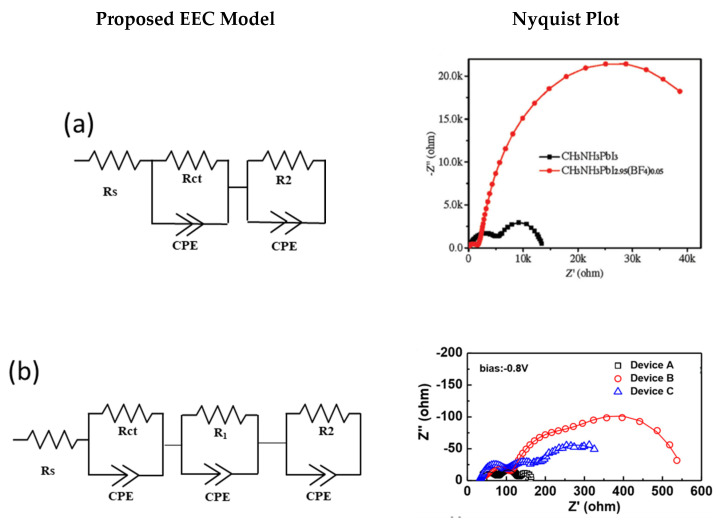

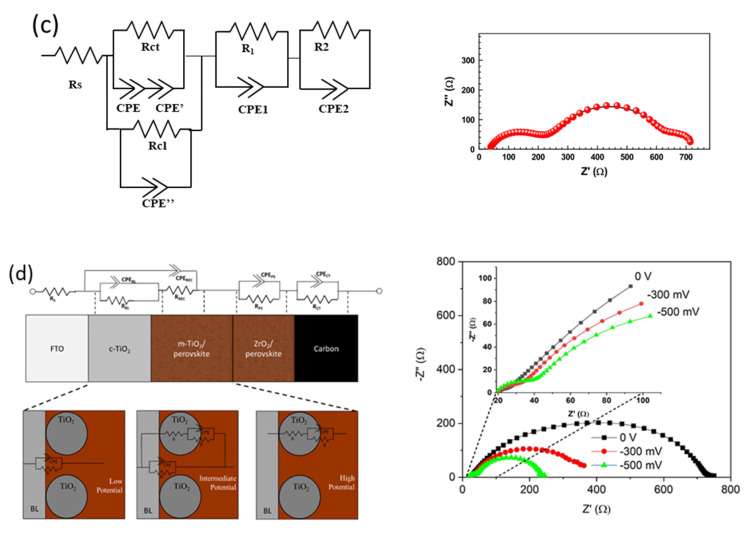
Electrical equivalent circuits for HTM-free perovskite solar cells with their corresponding Nyquist plots: (**a**) 2-RC model. Reproduced with permission from [32]. Wiley-VCH Verlag GmbH & Co. KGaA Weinheim, 2016. (**b**) Nyquist plots of Device A (containing MAPbI3 perovskite), Device B (containing MAPbI2.7Br0.3 perovskite), and Device C (containing MAPbI2.4Br0.6 perovskite), fitted with 3-RC model. Reproduced with permission from [30]. Royal Society of Chemistry, 2016. (**c**) Quadruple mesoscopic layer architecture fitted with 4-RC model. Reproduced with permission from [31], Elsevier, 2015. (**d**) Electric current follows the diagram for m-PSCs under low, intermediate, and high potentials. Reproduced with permission from [67]. Elsevier, 2020.

Various EECs have been used in previous works to model EIS spectra of HTM-free PSCs. For instance, Chen et al. [32] employed a 2-RC circuit model for a fully printable HTM-free PSC device with mixed anion perovskite at 0.6 V biasing, as illustrated in Figure 6a. Two arcs can be clearly differentiated in the Nyquist plot, and hence employing a two-circuit model seems logical. On the other hand, Cao et al. [30] used a 3-RC circuit model for a double-layer mesoscopic HTM-free PSC device at 0.8 V biasing. In this case, three arcs can be clearly separated in the Nyquist plot illustrated in Figure 6b. Therefore, employing a 3-RC circuit model for this case is also justifiable. Both models have been used consistently in other works for double-layer mesoscopic architectures [25,29,32,33,34,35,41,42,43,44,66]. The common reasoning provided for employing 2-RC elements in previous works is that, since the HTM layer is not present in the device, perovskite plays both roles of light-harvesting and hole transporting and, therefore, only two RC elements representing carbon/perovskite interface and perovskite/ETL interface are required to fit the EIS data. On the other hand, employing the 3-RC model is theoretically more reasonable since it considers an RC element for the mesoporous layers as well, in the EEC model. In some works, the third RC element is attributed to the mesoporous oxide layer/perovskite interface in the intermediate frequency region of the Nyquist plot [31], while, in others, it is assigned to ion motion [31,44] and slow dynamics in perovskite [30], represented in the low frequency (LF) region of the Nyquist plot. Zhou et al. [44] reported a 3-RC model for the fitting of a double-layer mesoscopic architecture under an applied biasing of 0.2 V, as illustrated in Figure 6b. However, it can be clearly observed that the EEC fitting of the Nyquist plot does not seem perfect. Raminafshar et al. [28] reported two models for different biasing conditions; a 2-RC circuit model for a double-layer mesoscopic HTM-free PSC device at 0 V biasing, whereas a 4-RC circuit model at 0.8 V biasing based on best fitting. One RC element was assigned to the high frequency arc representing charge transfer at counter electrode/perovskite interface and one RC element was attributed to the low frequency arc representing charge accumulation at the perovskite/ETL interface. The reasoning for the remaining two RC elements in the 4-RC circuit model was not clearly explained, and it was attributed to the appearance of additional interfacial charge accumulation under strong biasing. Cao et al. [31] employed a 4-RC circuit model to fit the EIS data of a triple-layer mesoscopic HTM-free PSC, as illustrated in Figure 6c. The additional RC element from the FTO or compact TiO_2_ layer was suggested to be omitted at high potentials due to a very high resistance and low capacitance value.

However, Ahmed et al. [67] performed a more detailed EEC-based EIS study for monolithic PSCs, where multiple EEC models were analyzed, and a well-defined model was recognized, which can be further modified based on the applied potentials (see Figure 6d). Monolithic PSCs (m-PSCs) were fabricated with a double mesoporous scaffold, m-TiO_2_ and ZrO_2_, and its EIS analysis was performed at high, intermediate, and low potentials. The 3-RC model was proposed to fit the EIS data at low and high potentials, and its authenticity was verified by comparing it with 2-RC and 4-RC models. The 3-RC model was chosen based on its relevancy to physical parameters of m-PSC, closeness to the experimental EIS data, and goodness of fit. The first RC element related to the HF region was attributed to the carbon/perovskite interface, which is the commonly accepted interpretation. The RC elements related to IF were attributed to the spacer layer, ZrO_2_, and mesoporous TiO_2_ contribution since the perovskite closely packs the mesoporous oxide particles. The RC element of the LF region was assigned to the recombination resistance at the perovskite/TiO_2_ interface. Moreover, the 3-RC model was suggested to be modified to 4-RC at intermediate potentials due to multiple contributions from perovskite/compact TiO_2_ and perovskite/mesoscopic TiO_2_ interfaces. At high potentials, the contribution from the compact layer can be ignored due to very high resistance and small capacitance. A current follow model, illustrated in Figure 6d, was developed at different potentials so that the EEC model could be modified according to the applied to bias.

From this literature review, it is clear that the interpretation of the IF and LF region is complex and has still not been widely agreed upon. Moreover, it is clear that the EEC model selection also depends on applied biasing in addition to the device architecture.

## 5. Analysis of HTM-Free PSCs with the EIS Technique

EIS has been utilized in HTM-free PSC analysis to compare different architectures [31], different compositions [29], and different thicknesses of the layers [43] by studying their interfacial properties. Below, we describe some of the ways that researchers have adopted to utilize the EIS tool in their studies.

### 5.1. Use of EIS for the Comparison of HTM-Based and HTM-Free PSCs

**Figure 7:**
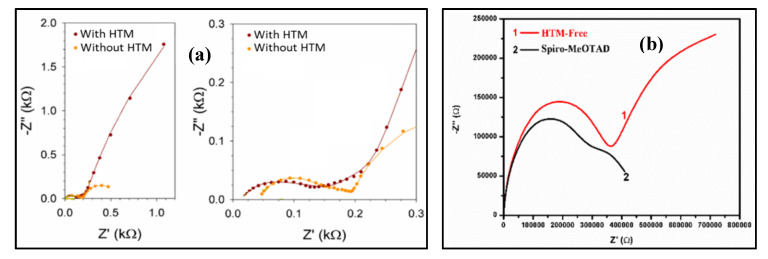
Nyquist plot with HTM and HTM-free carbon counter electrode-based PSCs: (**a**) obtained at a DC bias of 0.1 V under 1 sun illumination (Reproduced with permission from [25]. American Chemical Society, 2014.); and (**b**) obtained at DC bias of 0 V in the dark [28].

The first article employing the EIS tool to analyze HTM-free PSCs was demonstrated by Juárez-Pérez et al. [25] in 2014. They compared the effect of removing the HTM layer on the performance of the PSCs. Using the EIS (at 0.1 V biasing), the values of series resistance (R_s_), recombination resistances (R_rec_), and charge transfer resistance (R_ct_) (see Figure 7a) and their effect on V_oc_, fill factor (FF), and PCE can be determined. Raminafshar et al. [28] also performed EIS analysis at different biasing (at 0 V, as well as close to open-circuit voltage) in dark and illumination to compare carbon-based monolithic HTM-free PSCs with the effect of HTM on PSCs. Their EIS analysis has shown that the absence of an HTM layer causes a higher electrons holes recombination (see Figure 7b) due to the lack of an electron blocking effect at the perovskite/counter electrode interface. Moreover, the FF also decreases due to the higher R_s_ value in the absence of HTM. Similarly, Khan et al. [68] also performed a study on the impact of HTM layer in PSCs by EIS technique which also showed a lower recombination resistance in the case of HTM-free PSC devices.

### 5.2. Use of EIS to Identify the Impact of ETL and Mesoporous Scaffold

The properties of ETL and mesoporous oxide layers have a significant impact on charge transfer and recombination processes. The better is the energy alignment of ETL with perovskite, the lower is the recombination rate. The spacer layers, ZrO_2_ or Al_2_O_3_, play a crucial role in separating the front and back electrodes to prevent ohmic shunts [69]. The pore size determines the infiltration of perovskite and its contact with the anode. Therefore, the spacer layer thickness needs to be optimized to deliver high performance. Researchers have extensively utilized EIS tool to analyze the interfacial properties of HTM-free PSCs, by exploring different ETLs and spacer layers, and varying the mesoporous scaffold thickness, to achieve high-performance HTM-free PSCs, suitable for commercialization.

**Figure 8:**
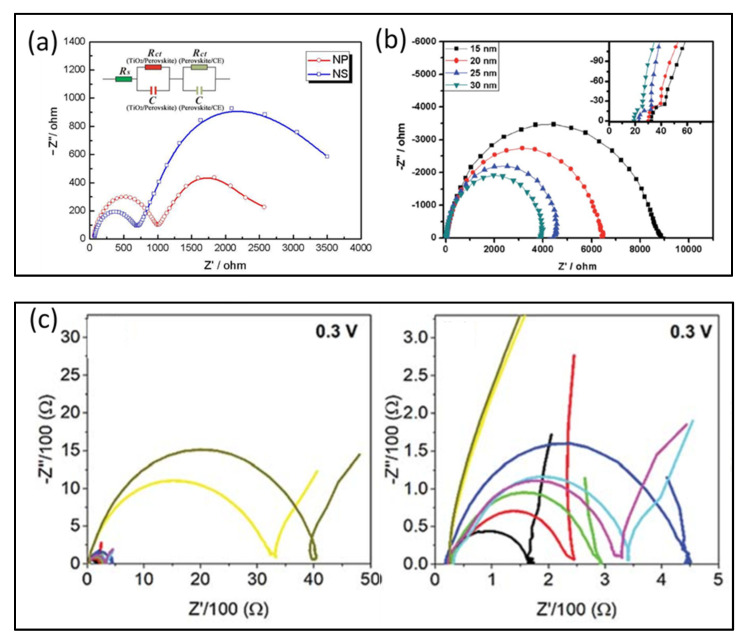
Nyquist plots of carbon-based monolithic HTM-free PSCs: (**a**) with TiO_2_ nanosheets and TiO_2_ nanoparticles, measured at 0.6 V in the dark (Reproduced with permission from [40], American Chemical Society, 2014.); (**b**) with a different thickness of TiO_2_ mesoporous layers, measured at 0.7 V in the dark (Reproduced with permission from [42]. Royal Society of Chemistry, 2015.); and (**c**) with different spacer layer thickness (0 μm (black), 0.08 μm (red), 0.11 μm (green), 0.66 μm (purple), 2.64 μm (light blue), 3.31 μm (dark blue), 4.84 μm (yellow), and 5.96 μm (black with yellow shade), measured at 0.3 V under 0.1 sun illumination (Reproduced with permission from [70]. Royal Society of Chemistry, 2014).

Hang’s group fabricated an HTM-free mesoscopic PSC and demonstrated a PCE of 10.64% [40]. TiO_2_ nanosheets (NSs) as an electron transport layer (ETL) was compared with TiO_2_ nanoparticles (NPs), and the EIS data showed better interfacial properties of the device with NSs. The Nyquist plot showed two well-defined arcs and the device with NSs provided a smaller arc in the HF region and a bigger arc in the low frequency (LF) region, indicating a smaller R_ct_ at the counter electrode/perovskite interface and a higher R_rec_ at the perovskite/ETL interface, compared to NPs and hence improving the performance (Figure 8a). The explanation provided was that the attachment between the perovskite and TiO_2_ nanosheets is stronger, and the high ionic charge of the facets in TiO_2_ nanosheets screens electrons, which results in lower recombination of electrons and holes. The EIS tool was also used to study the effect of different sized mesoporous TiO_2_ nanoparticles by analyzing charge transfer kinetics at the TiO_2_/perovskite interface [42]. It was observed that different sizes did not have an evident effect on the charge transfer at the selective contact represented by the HF arc, as illustrated in Figure 8b; however, the series resistance value was observed to be decreasing with an increase in the size of TiO_2_ nanoparticles. The low-frequency arc was assigned to interfacial or bulk recombination. The recombination resistance was observed to be decreasing with the increase in the size of TiO_2_ nanoparticles. This EIS analysis helped identify the optimum size of TiO_2_ nanoparticles; 25 nm, which exhibited the best performance with a PCE of 13.41%. Recently, Han’s group performed another EIS study under weak illumination (0.1 sun) on HTM-free mesoscopic PSC with a TiO_2_/spacer/carbon architecture and analyzed the spacer layer thickness to optimize the performance of the device [70]. The Nyquist plot obtained is illustrated in Figure 8c: it was different from the previous works, and an additional feature was observed; two RC elements were merging in the HF region, and one RC element was observed in the LF region. It was described that the additional RC element was due to charge transport of the perovskite in the spacer layer. To summarize, in the HF to intermediate frequency (IF) region, charge transport process was related to carbon/perovskite interface, together with perovskite/spacer layer, and the LF region was related to TiO_2_/perovskite interface. To confirm the reported correlations of each semicircle with the physical processes at the interfaces, the EIS study was also performed with and without the mesoporous TiO_2_ layer.

**Figure 9:**
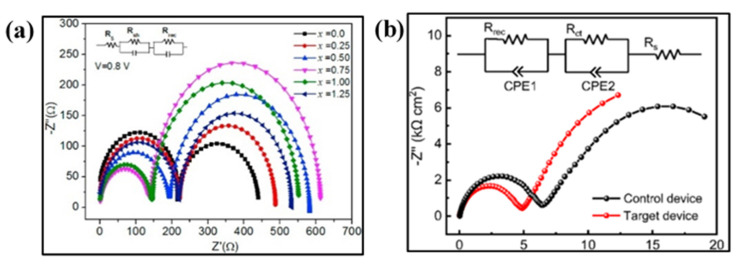
(**a**) Nyquist plots with different DEA molar ratios (*x*), measured at a bias of 0.8 V in the dark. Reproduced with permission from [71]. Elsevier, 2018. (**b**) Nyquist plots of HTM-free monolithic PSC with SnO_2_ as ETL (Control device) and bi-layered ZTO as ETL (Target device). Reproduced with permission from [66]. American Chemical Society, 2019.

Cao et al. [31] performed the EIS analysis of an efficient (with 15% efficiency) HTM-free PSC, with a quadruple mesoscopic layer, to determine its charge transfer processes in the range of 0.02 Hz to 2 MHz under illumination as well. It was observed that the Nyquist plot arcs shifted to high-frequency regions under illumination, giving crucial information on the carrier lifetime and changes in electron–hole density due to photoexcitation and, hence, providing a more realistic analysis under practical operating conditions (see Figure 6c). Liu et al. [71] investigated the effectiveness of compact TiO_2_ blocking layer in HTM-free PSCs, by optimizing the amount of DEA (diethanolamine) in the precursor solution, through EIS analysis. It was stated the conventional fabrication procedure of TiO_2_ is prone to cracking, which can create trapping sites for electrons and holes recombination. Consequently, the incorporation of DEA in the precursor solution revamps the cracks resulting in a higher performance of the PSCs by increasing the contact area. The EIS analysis confirmed the optimum DEA molar ratio (*x*) of 0.75. The Nyquist plot, as illustrated in Figure 9a, showed that the R_ct_ kept decreasing by increasing the amount of DEA until *x* = 0.75, where R_ct_ value was at its minimum. Any further increase in the DEA amount leads to an increase in R_ct_ value. Consequently, the R_rec_ increased to a maximum at *x* = 0.75 followed by a decrease in further increasing the value of *x* (Figure 9a). Later on, Zhao et al. [66] studied a bilayer zinc tin oxide (ZTO) film as an ETL instead of the commonly employed TiO_2_ or SnO_2_. Its EIS analysis shows that the ZTO performed better in terms of suppressing the recombination process of electrons and holes due to better-matched energy alignment of ZTO with the perovskite film, which enhances the V_oc_. This was proved by EIS. Moreover, the R_s_ and R_ct_ of ZTO-based PSC devices were reduced due to the higher charge mobility and conductivity, as shown in Figure 9b.

### 5.3. Use of EIS to Investigate the Hole Collecting (Counter) Electrodes

Hole collecting layers or counter electrodes (CEs) play a crucial role in determining the performance of PSCs. In HTM-free PSCs, it is required for the CE to play the role of HTM as well [72], which in turn affects the series resistance and V_oc_ of the device [35]. Carbon is most commonly employed as the CE in HTM-free PSCs, and an optimized thickness of mesoporous carbon CE is essential for high-performance devices (specifically, in the case of monolithic PSCs) [73]. Batmunkh et al. [74] suggested that carbon nanomaterials and graphene should be especially considered for CEs, as they play an essential role in significantly enhancing the stability of PSCs. Meng et al. [75] reviewed the interfacial engineering techniques of carbon-based PSCs and some of the modifications suggested in carbon paste as the CE, essential for enhancing the stability of PSCs, optimizing the thickness of the CE layer, hot-pressing, and increasing the contact sites. The EIS tool has been consistently utilized in analyzing the performance of CEs in HTM-free PSCs for determining their optimized thickness and compositions.

**Figure 10:**
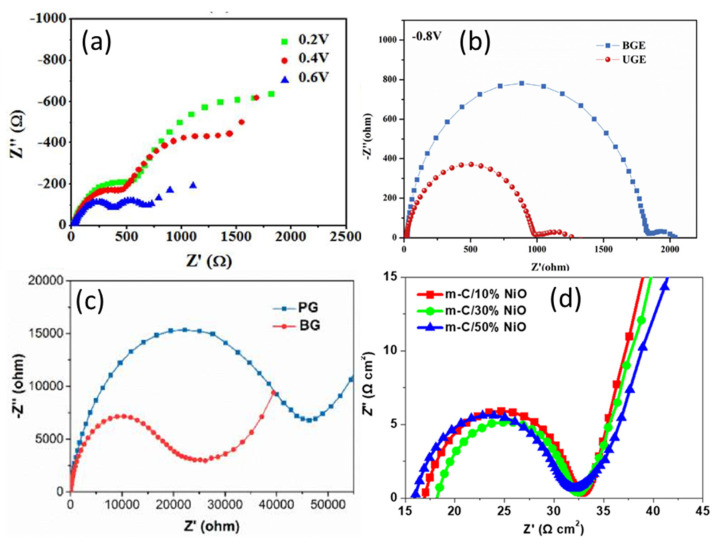
Nyquist plots of carbon counter electrode-based HTM-free PSCs: (**a**) graphite-based CE under different applied potentials (Reproduced with permission from [43]. Royal Society of Chemistry, 2013.); (**b**) with bulk graphite and ultrathin graphite CE, measured at 0.8 V in the dark (Reproduced with permission from [33]. Elsevier, 2017.); (**c**) with boron-free graphite and boron-doped graphite CE, measured at 0.6 V in the dark (Reproduced with permission from [34]. American Chemical Society, 2014.); and (**d**) with different amounts of NiO incorporated CE, measured at 0.5 V under illumination. Reproduced with permission from [35]. American Chemical Society, 2018.

Han’s group utilized the EIS tool to analyze the effect of the counter electrode (CE) on the photovoltaic performance of the fabricated HTM-free mesoscopic PSC device [43]. Graphite/carbon was used as the back-counter electrode, and the EIS technique analyzed the effect of the thickness of graphite in carbon counter electrodes in HTM-free PSCs at 0.2, 0.4, and 0.6 V DC biasing (see Figure 10a). It was observed that the graphite-based carbon electrode decreased the R_s_ and R_ct_ of the device and hence had a significant impact on increasing the PCE (up to 11%) of HTM-free cells. Next, they employed an ultrathin graphite-based carbon counter electrode for HTM-free mesoscopic fully printable PSC, analyzed its interfacial properties with the EIS tool, and compared it with bulk graphite counter electrode-based device, as shown in Figure 10b [33]. The analysis confirmed that the ultrathin graphite-based device exhibited better interfacial properties, higher charge transfer rate, and lower recombination rate due to its larger specific surface area and hence more contact surface with the perovskite increasing the hole transporting efficiency. Later on, they manipulated the counter electrode again, by boron doping of the graphite, and performed EIS analysis on it (see Figure 10c) [34], which showed that the charge transfer rate at the carbon/perovskite interface increased, and the charge recombination lifetime also extended. In another research, they analyzed the effect of the work function of the carbon counter electrode on the performance of mesoscopic HTM-free PSC [35]. They used the EIS tool to investigate the impact of incorporating NiO in the mesoporous carbon layer as the counter electrode and analyzed the charge transfer rate at the carbon/perovskite interface and recombination resistance at TiO_2_/perovskite interface as shown in Figure 10d.

**Figure 11:**
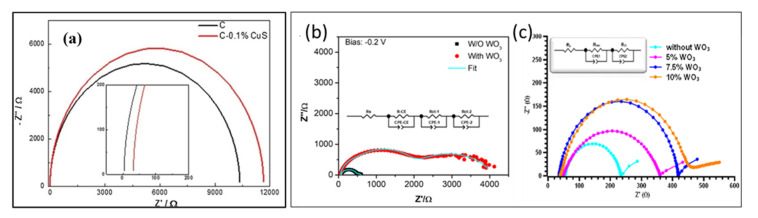
Nyquist plots of carbon counter electrode-based HTM-free PSC: (**a**) without and with 0.1% CuS incorporated carbon-based HTM-free PSCs (Reproduced with permission from [76]. Elsevier, 2018.); and (**b**,**c**) without WO_3_ additive and with WO_3_ additive. (**b**) Reproduced with permission from [44]. Royal Society of Chemistry, 2014. (**c**) Reproduced with permission from [29]. American Chemical Society, 2020.

Hu et al. [76] studied the effect of incorporating CuS in the carbon CE layer using EIS tool (see Figure 11a), and it was observed that the Rs value, which was claimed to be directly linked to the conduction properties of the metal electrode, decreased in CuS-C-based PSC. Moreover, the R_rec_, which was related to the recombination processes of the overall PCS, was reduced in CuS-C-based PSC, indicating better hole collection property of CuS-based carbon CE, as well as the better property of suppressing electrons and holes recombination, leading to an enhanced FF and Jsc. Zhou et al. [44] manipulated the carbon layer by embedding WO_3_ nanoparticles in it to function as the HTM. A PCE of 10.77% was achieved under ambient conditions. To analyze this HTM-free PSC, EIS analysis was performed in the range of 1 Hz to 1 MHz at −0.2 V DC biasing in the dark. As can be observed in Figure 11b, the HTM-free PSC with WO_3_ nanoparticles showed a lower R_s_ value compared to PSC without WO_3_, indicating higher hole extraction efficiency. Moreover, the R_rec_ was higher with WO_3_ embedded PSC, which means that the WO_3_ was acting as a passivating layer at the carbon and perovskite interface, inhibiting the recombination of charges. Later on, Bhandari et al. [29] also studied the effect of WO_3_ embedded carbon in HTM-free PSC with the EIS technique. This time, the EIS tool was used to optimize the amount of WO_3_ and EIS results of the best performing device; 7.5% WO_3_ showed a lower R_s_ value, which is directly correlated to the PCE and a higher R_rec_ at TiO_2_/perovskite interface (Figure 11c).

### 5.4. Use of the EIS to Investigate the Effect of Perovskite Composition

HTM-free PSCs are commonly composed of Lead halide-based perovskites [20,37] composition of the perovskite layer is crucial in determining the performance of HTM-free PSCs. The perovskite should deliver uniform pore filling to enhance the FF of the device. Moreover, it should also provide low defect concentration and high contact with the mesoporous scaffold in mesoporous HTM-free architectures. Additionally, an improved connection of perovskite with the CE is also required to suppress the recombination of electrons and holes and hence enhance the V_oc_. Researchers have extensively worked on optimizing the perovskite compositions in HTM-free PSCs and analyzed the photovoltaic performance using the EIS tool.

**Figure 12:**
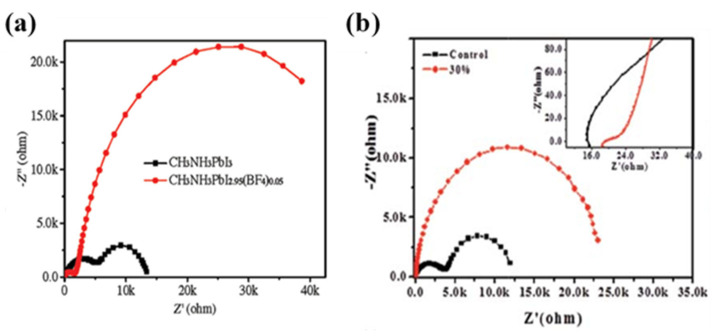
Nyquist plots of carbon counter electrode-based HTM-free PSCs: (**a**) with single anion and mixed anion perovskite, measured in the dark at a bias of 0.6 V (Reproduced with permission from [32]. Wiley-VCH Verlag GmbH & Co. KGaA Weinheim, 2015.); and (**b**) with perovskite incorporated with 30% LiCl and without it, measured in the dark at a bias of 0.6 V (Reproduced with permission from [41]. Royal Society of Chemistry, 2016.).

Han’s group fabricated HTM-free fully printable mesoscopic PSC with a mixed anion perovskite (CH_3_NH_3_PbI_(3−x)_(BF_4_)_x_) and used EIS tool to compare it with the single anion perovskite-based HTM-free PSC device [32]. The EIS results show that the transport and exchange resistance at the carbon/perovskite interface, represented by the HF arc, was smaller for the case of mixed anion perovskite-based device, resulting in an increased fill factor (FF) of the device. In addition, the recombination rate in the case of mixed anion perovskite-based device was much lower, as indicated by the larger arc in the LF region, resulting in an enhanced V_oc_ of the device, as illustrated in Figure 12a. They then manipulated the composition of perovskite again by incorporating 30% LiCl with it and achieved a PCE of 14.5% [41]. The EIS tool was used to analyze the interfacial properties, and it was observed that charge transfer resistance and recombination rate reduced dramatically (Figure 12b), resulting in an enhanced FF and V_oc_.

Later on, Cao et al. [30] also modified the perovskite precursor and analyzed the performance by EIS technique (see Figure 6b). MAPbI_3_-Br_x_ mixed perovskite precursor was fabricated, and a higher PCE than for other MAPbI_3_-based devices was obtained. The J–V curves showed reduced hysteresis, which was attributed to slow kinetics observed from impedance measurements. The hysteric effect was linked to low-frequency arcs in the Nyquist spectra, and hence it was concluded that the higher the low-frequency capacitance is, the higher the I–V hysteresis will be.

## 6. Summary and Future Perspectives

A wide variety of perspectives has been adopted by researchers to enhance the performance of the HTM-free PSCs; however, the primary focus was to explore the different counter electrode (CE) materials. Although the carbon-based HTM-free PSCs can simply be prepared using printing methods, the low power conversion efficiency (PCE) of ~15% is leftover as an essential research question. Besides, the challenge is still under debate to achieve consistent and high integrity on the consecutive layer and perovskite layer deposition for the massive scale production. The PCE can be observed to be decreasing with an increase in the area. More research work is required to further enhance its performance by exploring more optimization strategies. In addition, the widespread production of the HTM-free PSCs requires improving the device stability, deep understanding of the interfaces between the different printed layers, and charge collection at the respective electrodes.

In this respect, electrochemical impedance spectroscopy (EIS) has emerged as a leading-edge tool for a systematic illustration of the charge accumulation, charge transfer, interfaces analysis, and degradation of the different layers in the PSCs research. It is widely utilized by researchers to study the behavior of HTM-free PSCs under various operating conditions. Even though EIS has proven to be an advanced tool, with the help of which the interfacial and bulk properties of PSCs can be comprehensively analyzed, many aspects of this tool are yet to be fully understood, for example, identifying the correlation of each interface of the multilayered HTM-free PSC with the different features in each frequency range of the Nyquist plot. Many researchers still do not consider the charge transport processes at the spacer layers (nine in the case of monolithic design), ZrO_2_ or Al_2_O_3_, which are responsible for the additional features in the Nyquist plot. The charge transport processes from the perovskite confined in these spacer layers produce an additional feature in the Nyquist plot, which is commonly merged with the high-frequency arc and, therefore, is challenging to identify. In this respect, a well-supported electrical equivalent circuit (EEC) model can be developed to improve the accuracy of the analysis and hence can provide valid, relevant physical parameters. It is essential to acquire an in-depth understanding of EEC modeling of the impedance spectra since the underlying assumptions and applicability of this approach remains unclear in the literature.

It has been realized by EIS analysis that the key to optimizing the performance of HTM-free PSCs is by choosing an ETL with the following properties: provides high contact area to minimize trapping sites for recombination and well-matched energy alignment with perovskite. The combination of these properties results in reduced R_ct_ and R_s_, as realized by EIS analysis. The recombination process is also significantly suppressed. Besides, it has been found that the charge transfer resistance in the HF region increases with the spacer layer thickness. Therefore, it is crucial to optimize the spacer layer to obtain the high efficiency of HTM-free PSCs. In addition, it has been recognized (by EIS analysis) that mixed anion perovskites such as MAPbI_(3−x)_Br_x_ provide better performance by suppressing recombination rate and hence enhancing the V_oc_ of the device. This is attributed to their wider bandgap, which makes them capable of acting as electron blocking materials at the perovskite/counter electrode interface, and hence this avoids the requirement of an HTM layer.

The EIS study of the HTM-free PSCs has also proven that the hole collecting layer (counter electrode) indeed has a significant effect on HTM-free PSCs performance. The hole collecting layer is highly responsible for the HF region of the Nyquist plot. Moreover, it also affects the LF region of EIS spectra (in the case of monolithic architecture) because it provides a pathway to perovskite infiltration. Therefore, the better the porous morphology (absorbency) of CE is, the more uniform the perovskite infiltration will be in the monolithic design. Moreover, EIS analysis has also shown that manipulating the work function of the counter electrode to improve the energy alignment and enhance the conduction properties by doping is an effective technique in obtaining high-performance HTM-free PSCs.

We believe that the use of the EIS tool in the field of PSCs is essential, and utilizing this tool to its maximum potential will pave the way for substantial improvements in our understanding. EIS tool has been used consistently in previous works for the analysis of HTM-free PSCs, yet the scope for further studies is significant. The studies that are highlighted in this review can help researchers gain an intimate understanding of the interpretation of EIS data, and the different ways in which the EIS tool can be utilized to analyze HTM-free PSCs.
